# The Role of Astrocytes in the Modulation ofK^+^-Cl^−^-Cotransporter-2 Function

**DOI:** 10.3390/ijms21249539

**Published:** 2020-12-15

**Authors:** Tomoya Kitayama

**Affiliations:** School of Pharmacy and Pharmaceutical Sciences, Mukogawa Women’s University, Hyogo 663-8179, Japan; tomokita@mukogawa-u.ac.jp; Tel.: +81-(0)798-45-9967

**Keywords:** astrocyte, K^+^-Cl^−^-cotransporter-2, neuropathic pain, brain-derived neurotrophic factor, tropomyosin receptor kinase B

## Abstract

Neuropathic pain is characterized by spontaneous pain, pain sensations, and tactile allodynia. The pain sensory system normally functions under a fine balance between excitation and inhibition. Neuropathic pain arises when this balance is lost for some reason. In past reports, various mechanisms of neuropathic pain development have been reported, one of which is the downregulation of K^+^-Cl^−^-cotransporter-2 (KCC2) expression. In fact, various neuropathic pain models indicate a decrease in KCC2 expression. This decrease in KCC2 expression is often due to a brain-derived neurotrophic factor that is released from microglia. However, a similar reaction has been reported in astrocytes, and it is unclear whether astrocytes or microglia are more important. This review discusses the hypothesis that astrocytes have a crucial influence on the alteration of KCC2 expression.

## 1. Introduction

Neuropathic pain is characterized by spontaneous pain, pain sensations, and tactile allodynia. The pain sensory system normally functions under a fine balance between excitation and inhibition. Neuropathic pain arises when this balance is lost for some reason. In past reports, various mechanisms of neuropathic pain development have been reported, one of which is the downregulation of K^+^-Cl^−^-cotransporter-2 (KCC2) expression [1]. An intracellular chloride concentration is maintained by cation–chloride cotransporters, including KCC2, which is the potassium chloride exporter in neurons [2]. The alteration of intracellular chloride perturbs the balance between excitation and inhibition [1]. γ-aminobutyric acid (GABA), a typical inhibitory neurotransmitter, acts as an excitatory signal in the fetal brain [3]. During this period, KCC2 is expressed at low levels [4]. The GABAergic signal shifts from excitatory to inhibitory due to an increased KCC2 expression after birth. The intracellular chloride concentration is low, and this is maintained by KCC2. In this case, hyperpolarization is induced by the binding of an inhibitory neurotransmitter to the receptor, followed by an inflow of chloride ions via the chloride ion channel. An increase in the intracellular chloride concentration is induced by KCC2 downregulation. After reduction of the chloride gradient, depolarization is induced by the binding of an inhibitory neurotransmitter to the receptor, followed by an outflow of chloride ion via the chloride ion channel.

The downregulation of KCC2 is induced by various neuropathic pain models. The first reported instance of a relationship between neuropathic pain and the downregulation of KCC2 was in a rat model of peripheral nerve injury induced by implantation of a polyethylene cuff around the sciatic nerve [1]. Subsequently, our laboratory and others demonstrated that a sciatic nerve injury induced by other methods exhibited similar results [5,6,7,8]. The transection of the inferior alveolar nerve could induce neuropathic pain in the orofacial region and decrease KCC2 expression in the trigeminal spinal subnucleus caudalis [9]. In addition, several studies demonstrated that the downregulation of KCC2 was observed in chronic inflammatory pain models. The intraplantar injection of formalin [10] and complete Freund’s adjuvant [11] reduced the KCC2 expression in the spinal cord. A pulpal inflammation induced a decrease in the KCC2 mRNA level in the trigeminal subnucleus caudalis [12]. Allodynia was observed in a diabetes model animal induced by systemic streptozotocin administration, which also showed decreased KCC2 expression in the spinal cord [13]. Furthermore, this author demonstrated a relationship between KCC2 and allodynia by pharmacological methods. Naïve rats injected with R-(+)-((dihydroindenyl)oxy) alkanoic acid (R-DIOA), a KCC2 inhibitor, exhibited allodynia, similar to diabetic rats. These reports suggested that neuropathy induced by nerve injury and various inflammation resulted in chronic pain via KCC2 downregulation in neurons.

Coull et al. first reported the instance of a relationship between neuropathic pain and the downregulation of KCC2 [1]. They demonstrated that the brain-derived neurotrophic factor (BDNF) induced the downregulation of KCC2, a response that contributed to the development of neuropathic pain-related allodynia and hyperalgesia [14]. It is believed that microglia were responsible for BDNF release. Many other reports have indicated a relationship between microglia and neuropathic pain (see, for review, [15,16,17,18,19,20]). However, recently, attention has been focused on the role of astrocytes in neuropathic pain. In this review is discussed the possibility that astrocytes play a key role in the modulation of KCC2 function.

## 2. Modulation of KCC2 Function by Several Cascade Reaction

BDNF has been shown to downregulate KCC2 expression by activating the tropomyosin receptor kinase B (TrkB). This cascade reaction has been suggested as one of the mechanisms of neuropathic pain development [14]. The BDNF-TrkB-KCC2 cascade reaction is most often investigated for the development of neuropathic pain. Consequently, there are many reports of molecular activity alterations that affect the BDNF-TrkB-KCC2 cascade reaction (Table 1). Existing evidence suggests that the BDNF-TrkB-KCC2 cascade reaction in neuropathic pain is sex-dependent [21]. However, there is no sex difference in the development of neuropathic pain induced by the direct inhibition of KCC2 in uninjured animals [22]. The KCC2 function may also be regulated by phosphorylation without the BDNF-TrkB-KCC2 cascade reaction. The KCC2 function can be regulated by direct phosphorylation [23,24]. In particular, the phosphorylation of the 940th serine of KCC2 leads to the enhancement or stabilization of KCC2 expression in the plasma membrane of neurons [25]. This phosphorylation is regulated by the group 1 metabotropic glutamate receptors [26]. Similarly, the stimulation of other G protein-coupled receptors can regulate KCC2 activity (Table 1). Recent studies have reported the epigenetic regulation of KCC2 expression but only in complete Freund’s adjuvant-induced inflammation pain models [27].

## 3. Astrocytes Maintain Balance of the Sensory System between Excitation and Inhibition

Astrocytes contribute to the maintenance of physiological levels of extracellular potassium ions, glutamate, glycine, and water in the absence of a pathology [44,45,46,47]. KCC2 is a potassium ion-dependent chloride exporter. The function of KCC2 is dependent on the difference in potassium ion concentration inside and outside of the neuron. In order for KCC2 to function, it is necessary to keep the extracellular potassium ion concentration at low levels, and astrocytes are important for maintaining this low-level concentration of potassium ion. 

Glycine serves as a major inhibitory neurotransmitter in the spinal cord. Glycine signals are directly involved in neuropathic pain [48]. The concentration of extracellular glycine is regulated mainly by two types of glycine transporters: glycine transporter 1 (GlyT1) and glycine transporter 2 (GlyT2), which are Na^+^/Cl^−^-dependent transporters. GlyT1 is widely expressed in the glia [46], and GlyT2 is largely localized to the presynaptic terminals of glycinergic neurons [49]. Since GlyT2 is expressed in the glycine nerve, it mediates the clearance of postsynaptically released glycine at the inhibitory synaptic cleft [50]. Several past reports have suggested the role of GlyT1 in lowering the extracellular glycine concentration [45,46]. Low extracellular glycine levels prevent the saturation of the glycine binding site on the *N*-methyl-*D*-aspartate (NMDA) receptor, an ionotropic glutamate receptor. The NMDA receptor increases the cation permeability by binding to glutamate, but its activation requires the binding of glycine to the NR1 subunit [51]. GlyT1 reduces the activity of excitatory synapses. In contrast, the inhibition of GlyT2, as well as GlyT1, strengthens the glycine receptor systems and suppresses neuropathic pain [8]. 

Glutamate serves as major excitatory neurotransmitter in the central nervous system. Glutamate is cleared from the excitatory synaptic cleft by astrocytes via glutamate transporters, which are then converted into glutamine and then released and, in turn, taken up by neurons [52]. The modulation of extracellular glutamate concentration directly affects neuropathic pain. The ligation of the sciatic nerve induces the downregulation of glutamate transporters in astrocytes at the spinal dorsal horn [53]. The dysfunction of glutamate transporters induces an increase in extracellular glutamate levels, leading to the development of pain [54]. The change in the extracellular glutamate concentration directly affects excitatory signaling and, also, affects the function of KCC2. KCC2 functions are regulated by group 1 metabotropic glutamate receptors via the phosphorylation of KCC2 [26]. The phosphorylation of KCC2 occurs downstream of protein kinase C (PKC). The state of the NMDA receptor activity is closely related to the KCC2 function [32,33]. The NMDA receptor function is regulated by extracellular glycine levels. These reports indicate that transporters expressed in astrocytes play an important role in maintaining the function of KCC2 and in the development of neuropathic pain. 

## 4. Astrocytes Release Proinflammatory Cytokines and Chemokines after Injury

Inflammatory stimuli activate astrocytes and induce the activation of intracellular kinases and transcription factors. For example, a treatment with lipopolysaccharide leads to c-Jun *N*-terminal kinase/p38 mitogen-activated protein kinase (MAPK) phosphorylation, which is important in the induction of proinflammatory gene expression in the central nervous system [55]. These authors also demonstrated that lipopolysaccharide exposure upregulates the nuclear translocation of nuclear factor-κB (NF-κB). Similarly, the increase of MAPK phosphorylation and upregulation of NF-κB nuclear translocation were observed in neuropathic pain models induced by nerve injury [56]. These signals are upstream of the synthesis and secretion of proinflammatory cytokines, such as tumor necrosis factor (TNF) and interleukin (IL), which affect KCC2 functions [6,35]. 

Kitayama et al. demonstrated that astrocyte-derived IL-6 plays an important role in the development of neuropathic pain via the modulation of KCC2 functions [6]. The expression of IL-6 and BDNF were increased, and KCC2 expression was decreased in the spinal cord of a mouse model of neuropathic pain. The elevation of BDNF expression was prevented by IL-6 knockdown via RNA interference. This suppression of BDNF expression blocked the reduction of KCC2 expression. In this model, IL-6 expression was suppressed by blocking NF-κB nuclear translocation and preventing the upregulation of BDNF expression. In short, astrocyte-derived IL-6 increases BDNF expression, which, in turn, reduces the neuronal KCC2 expression by activating the TrkB receptor. Similarly, IL-1β and tumor necrosis factor alpha (TNFα) are secreted by astrocytes and have been suggested to regulate the BDNF-TrkB-KCC2 cascade reaction [35,36]. The BDNF mRNA expression is reduced by the intrahippocampal IL-1β injection in hippocampus CA1, CA3, and dentate gyrus [57]. However, IL-1β administration increases BDNF expression in reactive astrocytes [58]. Similarly, TNFα leads to the upregulation of BDNF expression in the primary cultures of astrocytes [59]. These reports suggested that BDNF is released by astrocytes stimulated by proinflammatory cytokines, which, in turn, suppresses KCC2 expression.

Astrocytes secrete several chemokines, such as CCL2 and CXCL1, after a nerve injury [60]. Astrocyte-derived CCL2 and CXCL1 regulate NMDA receptor currents in the spinal cord [61]. The alteration of NMDA currents is extracellular signal-regulated kinase (ERK)-dependent. CCL2 induces rapid phospho-ERK activation and the potentiation of NMDA currents. As described above, the state of the NMDA receptor activity is closely related to KCC2 function [32,33]. CCL2 has also been reported to cause the disinhibition of spinal cord neurons by suppressing GABA-induced currents [62]. The disinhibition may be due to an increase in intracellular chloride ions via decreased KCC2 activity. The downregulation of KCC2 leads to a reduction of the anion-reversal potential in neurons [1,14]. These studies indicated that astrocyte-derived CCL2 and CXCL1 increase the excitatory postsynaptic currents (EPSCs) in the spinal cord after nerve injury. In particular, late-phase neuropathic pain is reversed by SB225002, an antagonist of CXCR2, which is a receptor for CXCL1 [63]. 

Several cytokine receptors are expressed on astrocytes [64]. Astrocytes are activated by proinflammatory cytokine signals and promote the nuclear translocation of NF-κB, which likely activates the production of proinflammatory cytokines and chemokines. This system may be an autocrine mechanism for maintaining the active state and upregulation of BDNF expression. However, it is highly possible that astrocytes will also respond to cytokine releases from the microglia. 

## 5. Astrocyte Modulates Purinergic Signaling

Astrocytes release ATP by a variety of molecular mechanisms such as exocytosis, gap junction hemichannels, and anion channels [65,66,67]. The release of ATP from astrocytes exocytosis accounts for a large proportion under physiological conditions [68]. In neurological disorders, astrocytes release ATP to the extracellular space by nonvesicular mechanisms, such as connexin (CX) hemichannels [69]. The gap junction of astrocytes is composed by CX43. Nerve injuries induce an increase in CX43 expression, and CX43 hemichannels can release cytosolic ATP into the extracellular space [70]. The reduction of ATP release from astrocytes in the spinal cord evoked by a spinal cord injury was observed in *Cx43*^−/−^ knockout mice compared with wildtype mice [71]. *Cx43*^−/−^ knockout reduced the neuroinflammation induced by a nerve injury, indicating that CX43 hemichannel-mediated ATP release evokes neuropathic pain. 

ATP is an agonist for the purinergic receptors P2X and P2Y, which modulate the function of various neurons and microglia [16,42,43,72,73,74]. For example, P2X receptors regulate GABA release, which serves as a major inhibitory neurotransmitter in the central nervous system [72,73]. P2X receptors modulate the function of NMDA receptors via the regulated expression of NMDA receptor subunits NR1 and NR2B [74]. There is a glycine-binding site in NR1, and the function of NR2B is closely related to the function of KCC2 [32,51]. These reports indicate that the P2X receptor may regulate KCC2 functions, as well as neurotransmissions in neurons via alteration of the NMDA receptor. 

Several purinergic receptors are expressed in the microglia. CX43 hemichannel-mediated ATP releases also serve as mediators of intercellular astrocyte–microglia cell interactions. These receptors, including P2X purinoreceptor 4 (P2RX4), P2RX7, and P2Y purinoreceptor 12, are upregulated in the spinal microglia and play a key role in the development of neuropathic pain (see, for review, [16]). The stimulation of P2RX4 upregulates BDNF synthesis and release in the microglia [42,43]. The enhancement of BDNF expression decreases KCC2 expression in neurons via the BDNF–TrkB–KCC2 cascade reaction. Therefore, the CX43-mediated ATP release induces BDNF secretion from the microglia by stimulating P2RX4, which, in turn, activates the TrkB receptor in neurons and suppresses KCC2 expression. 

Astrocyte-derived ATP is hydrolyzed in the extracellular space to produce adenosine. Interestingly, adenosine suppresses nociception via adenosine A receptor activation [28,75]. In particular, the activation of the adenosine A3 receptor suppresses the onset of pain in a chronic constriction injury-induced neuropathic pain model in which a decrease in KCC2 expression is observed. Adenosine A3 receptor agonists, such as MRS5698, induce rapid PKC activation and restore GABA signaling [28]. Adenosine A3 receptor-mediated modulation of the GABA signaling pathway is associated with an enhancement of KCC2 serine phosphorylation and a reduction in glutamate decarboxylase 65 and GABA transporter-1 serine dephosphorylation, which are induced by chronic constriction injury. The adenosine A3 receptor-mediated activation of PKC results in enhanced or stabilized KCC2 expression in the neuronal plasma membrane by phosphorylation of the 940th serine residue of KCC2 [28]. 

## 6. Upregulation of Matrix Metalloproteases in Astrocytes after Injury

Matrix metalloproteinases (MMPs) have a wide range of actions, such as decomposition of the extracellular matrix, consisting of collagen, proteoglycan, and elastin; decomposition of proteins expressed on the cell surface; and interactions with bioactive substances. MMPs are also widely involved in the inflammation and tissue remodeling associated with various types of neurodegeneration [76]. Notably, MMP2 and MMP9 are upregulated in the spinal cord after spinal nerve ligation [77]. While the expressions of MMP2 and MMP9 are both enhanced, their time courses and cells are different. MMP9 upregulation is rapid and transient in sensory neurons after nerve ligation. This upregulation peaks in one day and decreases three days after nerve ligation. The nerve ligation leads to early activation of the microglia, followed by a late but sustained MMP2 upregulation in spinal astrocytes. This upregulation is maintained for seven to 21 days after nerve ligation. MMP9 activation is not essential for neuropathic pain development. The MMP9 knockout mice show efficacy primarily in the early phase of neuropathic pain. In contrast, the inhibition of MMP2 is effective in attenuating both the early and late phases of neuropathic pain [77]. 

IL-1β is produced as a precursor protein and enzymatically cleaved by MMP2 and MMP9 to produce the active protein. Thus, the maintenance of neuropathic pain by both MMP upregulations after spinal nerve ligation is a response to IL-1β activation [77]. As described above, the treatment of IL-1β increases BDNF expression in astrocytes [58]. Both the MMP-mediated activation of IL-1β may induce BDNF secretion from astrocytes, which, in turn, activates the TrkB receptor in neurons and suppresses KCC2 expression. In addition, MMP2 and MMP9 also activate BDNF [37]. Therefore, both MMPs can also enhance the BDNF-TrkB-KCC2 cascade reaction by directly activating BDNF. 

## 7. The Alteration of Zinc Ion Signaling in Astrocytes and the Central Nervous System after Injury

Zinc is an essential trace mineral that has important roles in neurodegenerative diseases, including neuropathic pain [6,78]. Zinc ion levels are controlled by various zinc transporter (ZnT) proteins. In mice, 14 zip were shown to increase, whereas nine znt were shown to decrease the intracellular zinc levels [79]. Extracellular zinc ion concentrations reach high levels during pathological conditions, and the source of zinc ion are neurons [78,79]. In presynaptic terminals, zinc ions are stored in vesicles by ZnT3, and zinc ions are released from these vesicles after nerve injuries. 

High extracellular levels of zinc ions have various effects on other molecules, including the BDNF-TrkB-KCC2 cascade reaction. The active region of MMPs include zinc ions. Zinc ions meditate the extracellular activation of MMPs, including MMP2 and MMP9 [37]. The activation of both MMPs underlies the upregulation of BDNF maturation and IL-1β signaling [37,77]. This alteration promotes the BDNF-TrkB-KCC2 cascade reaction and reduces the KCC2 expression. A high concentration of zinc ions inhibits the transport of glutamate in primary cultures of astrocytes. This inhibition elevates the extracellular glutamate levels [78]. Excitatory amino acids such as glutamate exacerbate pain directly via neurotransmissions but, also, regulate KCC2 functions via the NMDA receptor [32,33]. Although these reports indicate the indirect effects on KCC2 downregulation via the activation of MMPs and the NMDA receptor, zinc ions upregulate KCC2 expression directly via the receptor. Zinc ion in the synaptic cleft stimulates the metabotropic zinc sensing receptor (mZnR). The activation of mZnR leads to a metabotropic calcium response, and the resultant downstream ERK1/2 activation upregulates the phosphorylation of the 940th serine residue of KCC2 that leads to the enhancement or stabilization of KCC2 expression in the plasma membrane of neurons, thereby inducing a hyperpolarizing shift in GABA reversal potential [39]. However, it should be noted that this study was a hippocampal slice in the absence of pathology. 

Astrocytes uptake zinc ion under normal conditions and stressful conditions [80,81]. Oxidative stress increases the zinc ion uptake activity of astrocytes via the upregulation of Zrt/Irt-like protein (ZIP) 1 expression [81]. In contrast, partial sciatic nerve ligation induces the downregulation of ZnT1 expression in spinal astrocytes [6]. Therefore, astrocytes increase the ZIP1-mediated uptake of zinc ion and reduce ZnT1 expression, resulting in reduced zinc ion excretion under pathological conditions. The alteration is effective in suppressing the elevation in extracellular zinc ion levels but results in an increase in zinc ion levels in astrocytes. Elevated zinc ion levels in astrocytes cause a decrease in KCC2 expression [6]. The knockdown of ZnT1 by RNA interference leads to the elevation of intracellular zinc ion levels in primary spinal astrocyte cultures. The knockdown of ZnT1 upregulates NF-κB nuclear translocation and induces IL-6 maturation in primary spinal astrocyte cultures. IL-6 enhances BDNF expression through the activation of protein kinase A. The transplantation of ZnT1 knockdown astrocytes into the spinal cords of mice causes allodynia and the downregulation of KCC2 expression, both of which are suppressed by TrkB receptor inhibition. These studies suggest that elevated extracellular zinc ion levels lead to activation of the BDNF-TrkB-KCC2 cascade reaction mediated by activated astrocytes.

## 8. The Effects of KCC2 Downregulation Depends on the Region of Central Nervous System

Most of the reports so far have been on spinal cord alterations. Neuropathic pain also develops in the orofacial region, with reduced KCC2 expression in the trigeminal spinal subnucleus caudalis [9]. A masseter injury increases the expression of CX43 and IL-1β in reactive astrocytes at the spinal trigeminal nucleus [82]. The administration of P2X purinoreceptor 3 antagonists relieve facial pain induced by chronic constriction injury of the trigeminal infraorbital nerve [83]. The ligation of the infraorbital nerve induces chemokines and p38 MAPK upregulation, which modulate trigeminal neuropathic pain via the TNF and IL-1β signaling [84]. These studies indicate that the activation of astrocytes in the trigeminal nucleus appears to share many features with spinal astrocytes. Tornberg et al. reported an experiment using KCC2-deficient mice in the brain [85]. This experiment indicated that KCC2-deficient mice reduced in sensitivity to tactile and noxious thermal stimuli. The reason is unclear, but it may be related to the facilitation of the descending pain inhibitory system. In the absence of pathology, the descending pain inhibitory system is suppressed by the GABA signal in the periaqueductal gray. 

## 9. Discussion

This review discusses the importance of astrocytes in the development of neuropathic pain. In addition, the astrocyte regulation of KCC2 expression is involved in one of mechanisms of neuropathic pain development. Astrocytes modulate KCC2 expression by secreting proinflammatory cytokines and BDNF. These factors activate the BDNF-TrkB-KCC2 cascade reaction and suppress KCC2 expression in neurons. However, a similar reaction has been reported in the microglia, and it is unclear whether astrocytes or microglia are more important. 

Moy et al. reported an experiment using transgenic mice in which microglia cannot synthesize BDNF [21]. This experiment suggested that microglia are not essential as a source of BDNF for developing neuropathic pain. Our laboratory demonstrated that the knockdown of ZnT1 leads to the upregulation of BDNF expression in primary cultures of astrocytes. A ZnT1 knockdown astrocyte-conditioned medium induced allodynia [6]. However, the duration of action of the conditioned medium was shorter than the model of a peripheral nerve injury. Therefore, BDNF from both the microglia and astrocytes may activate the BDNF-TrkB-KCC2 cascade reaction in parallel and suppress the neuronal KCC2 expression. In contrast, proinflammatory cytokines, such as IL-6, IL-1β, and TNFα, are likely to function as a mediator of intercellular astrocyte–microglia cell interactions.

Both the initiation and duration of activation after an injury also differs between the microglia and astrocytes. Microglia rapidly respond and proliferate and reach the maximal levels until seven days after a peripheral nerve injury [15]. This reaction returns to normal levels within three weeks. In astrocytes, astrogliosis occurs one week after a nerve injury and continues for several months [86]. Thus, while the exact temporal cause of the disease depends on individual studies and injury methods, microglial activation usually precedes astrocyte activation. KCC2 expression is inhibited by BDNF released from the microglia in the first week after a nerve injury. Subsequently, this is maintained by BDNF released from astrocytes. Similarly, proinflammatory cytokines are rapidly secreted by microglia, which induce astrocyte activation. Microglia inhibitors are primarily only effective in the early phase of neuropathic pain [87]. By contrast, the inhibition of astrocytes is effective in attenuating both the early and late phases of neuropathic pain [63,87]. These studies suggest that microglia may play a role in exacerbating early-stage pain, and this may also apply to the regulation of KCC2 expression. 

Rapid microglia activation after a nerve injury is mediated by the extracellular release of various substances, including ATP. ATP released from astrocytes activate the microglia in the early phase after a nerve injury. This activation of microglia is suppressed by treatment with connexin channel inhibitors in the central nervous system [88]. A nerve injury via the CX43-mediated ATP release from astrocytes activates the microglia, enhances BDNF release from the microglia, and stimulates TrkB receptors in neurons, all of which downregulate KCC2 in the early phase of neuropathic pain. 

KCC2 downregulation induces chloride reversal potentiation in the spinal neurons in animal models of a neuropathic pain model [1]. This alteration causes an inhibitory neurotransmission that induces hyperpolarization to a lesser degree and, paradoxically, depolarizes neurons [14,89]. In other words, GABA and glycine signals do not suppress pain and may instead promote pain development in neuropathic pain models. However, the activation of glycine signals has been shown to ameliorate symptoms of neuropathic pain a few days after nerve injury [8], and these effects lasted for at least a few months [8]. While the effects of inhibitory neurotransmission depend on the individual studies and injury methods, the amelioration of pain by glycine signals has been reported in a sciatic nerve ligation model, streptozotocin diabetic model, and complete Freund’s adjuvant-induced inflammatory model [8]. In contrast, an intrathecal injection with GlyT inhibitors does not suppress neuropathic pain within three days after a nerve injury [8]. Interestingly, the intrathecal injection of strychnine, a glycine receptor inhibitor, ameliorates pain for three days after operation. Similarly, these reactions are also observed with GABA signals. The paradoxical depolarizations of inhibitory neurons by KCC2 downregulation occurs only during the development of neuropathic pain. Inhibitory signals are thought to function normally during the maintenance phase of neuropathic pain, regardless of KCC2 expression. These experiments indicate that KCC2 downregulation is not important during the maintenance phase of neuropathic pain but is important during the initiation and/or development phase [8,89]. During the neuropathic pain maintenance phase, astrocytes produce several bioactive substances that are involved in the regulation of KCC2 function, but these substances can have other effects during this period. For example, CCL2 and CXCL1 may indirectly regulate the function of KCC2, as described above. Both chemokines induced neuronal plasticity, resulting in an increase in NMDA receptor currents and the suppression of GABA-mediated transmissions [65,66]. This alteration leads to the potentiation of excitatory postsynaptic currents. Long-term central sensitization occurs because astrocytes remain active. Similarly, for the sustained activation of MMPs, it may respond differently to the regulation of KCC2 via the maturation of IL-1β. Other effects of IL-1β are outside the scope of this review and are covered in other excellent papers. Attention should be paid to the relationship between astrocytes and KCC2 functions in the early phase of neuropathic pain.

In the early phase of neuropathic pain, inhibitory neurotransmissions paradoxically induce the depolarization of neurons as a result of the chloride ion reversal potentiation induced by the downregulation of KCC2 expression in neurons [8,89]. This fact is important from the viewpoint of pain management, but the decrease in KCC2 expression not only induces pain but is also involved in the initiation and/or development of neuropathic pain [89]. The knockdown of KCC2 induced by the intrathecal injection of a small interfering RNA against KCC2 induces the rapid but transient downregulation of KCC2 expression, peaking at three days and recovering after seven days. KCC2 knockdown mice have been shown to develop allodynia that lasts for more a month [6].

**Figure 1:**
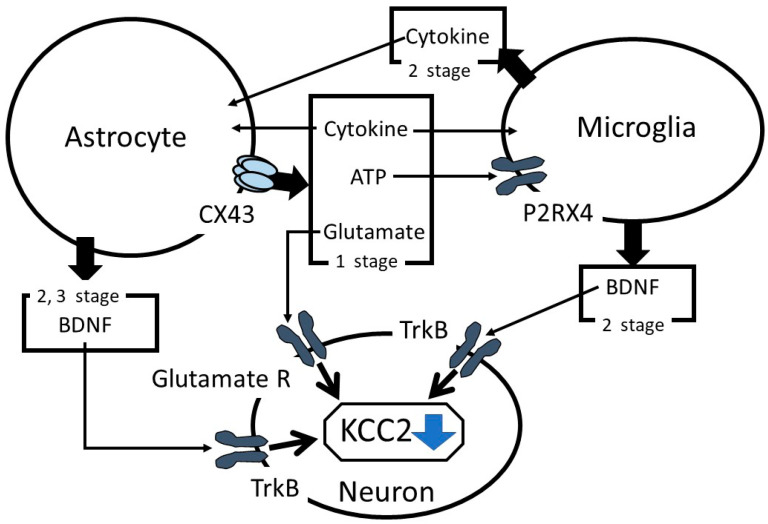
Summary of the role of astrocytes in the alterations of K^+^-Cl^−^-cotransporter-2 (KCC2) functions. TrkB: tropomyosin receptor kinase B and BDNF: brain-derived neurotrophic factor.

In summary, the role of astrocytes in suppressing KCC2 expression in neuropathic pain has been discussed. After a nerve injury, in the first stage, astrocytes release ATP and proinflammatory cytokines in a CX43-dependent mechanism, consequently activating the microglia and initiating astrocyte stimulation (Figure 1). Astrocyte CX43 mediates the release of glutamate, which regulates KCC2 functions via controlling the phosphorylation state of KCC2. In the second stage, the microglia are rapidly activated and suppress KCC2 expression via the BDNF-TrkB-KCC2 cascade reaction and, in parallel, promote astrocyte activation. In the third stage, proinflammatory cytokines released from the microglia and astrocytes stimulate astrocytes to increase BDNF production, which enhances the BDNF-TrkB-KCC2 cascade reaction. Therefore, astrocytes modulate KCC2 functions by various reactions and play an important role in the initiation and/or development of neuropathic pain.

## 10. Future Perspectives

Astrocytes play a key role in KCC2 downregulation in the initiation and/or development of neuropathic pain without a maintenance period. Astrocytes should be investigated in detail as an initiator of neuropathic pain. It is possible that control of astrocyte functions could prevent the development of neuropathic pain, as well as the maintenance phase. Our final goal is to prevent the development of neuropathic pain. Further analyses of astrocytes are needed for that purpose. 

## Figures and Tables

**Table 1:** Molecules that affect the K^+^-Cl^−^-cotransporter-2 (KCC2) function.

Molecule	Mechanisms	References
Adenosine receptor, A3	Phosphorylation control	Ford et al. [28]
Calcitonin gene-related receptor	BDNF-TrkB-KCC2 reaction	Buldyrev et al. [29]
Erythropoietin	BDNF-TrkB-KCC2 reaction	Jantzie et al. [30]
Glucocorticoid	BDNF-TrkB-KCC2 reaction	Luo et al. [31]
Glutamate receptor (group 1 metabotropic)	Phosphorylation control	Banke et al. [26]
Glutamate receptor (NMDA receptor)	Phosphorylation control	Hildebrand et al. [32] Lee et al. [33]
High salt loading	BDNF-TrkB-KCC2 reaction	Balapattabi et al. [34]
Interleukin-1β Tumor necrosis factor α	BDNF-TrkB-KCC2 reaction	Zelenka et al. [35] Souza et al. [36]
Interleukin-6	BDNF-TrkB-KCC2 reaction	Kitayama et al. [6]
Matrix metalloproteinase 2 Matrix metalloproteinase 9	BDNF-TrkB-KCC2 reaction	Hwang et al. [37]
Melatonin	BDNF-TrkB-KCC2 reaction	Wu et al. [38]
Metabotropic zinc sensing receptor (mZnR/GPR39)	Phosphorylation control	Chorin et al. [39]
Nicotinic acetylcholine receptor	BDNF-TrkB-KCC2 reaction	Gu et al. [40]
Opioid receptor (μ)	BDNF-TrkB-KCC2 reaction	Taylor et al. [41]
P2X purinergic receptor 4	BDNF-TrkB-KCC2 reaction	Trang et al. [42] Ulmann et al. [43]
Zinc transporter 1	BDNF-TrkB-KCC2 reaction	Kitayama et al. [6]

NMDA: *N*-methyl-*D*-aspartate, BDNF: brain-derived neurotrophic factor, and TrkB: tropomyosin receptor kinase B.

## Abbreviations

BDNF	Brain-derived neurotrophic factor
CX	Connexin
ERK	Extracellular signal-regulated kinase
GABA	γ-aminobutyric acid
GlyT	Glycine transporter
IL	Interleukin
KCC2	K^+^-Cl^−^-cotransporter-2
MAPK	Mitogen-activated protein kinase
MMP	Matrix metalloproteinase
mZnR	Metabotropic zinc sensing receptor
NF-κB	Nuclear factor-κB
NMDA	*N*-methyl-*D*-aspartate
P2RX4	P2X purinoreceptor 4
PKC	Protein Kinase C
TNF	Tumor necrosis factor
TrkB	Tropomyosin receptor kinase B
ZIP	Zrt/Irt-like protein
ZnT	Zinc transporter
